# Predictors and survival of cardiomyopathy determined by echocardiography in Thai patients with early systemic sclerosis: an inception cohort study

**DOI:** 10.1038/s41598-023-34110-1

**Published:** 2023-04-28

**Authors:** Suparaporn Wangkaew, Narawudt Prasertwitayakij, Jirapath Intum, Juntima Euathrongchit

**Affiliations:** 1 Division of Rheumatology, Department of Internal Medicine, Faculty of Medicine, Chiang Mai University, Chiang Mai, Thailand; 2 Division of Cardiology, Department of Internal Medicine, Faculty of Medicine, Chiang Mai University, Chiang Mai, Thailand; 3 Division of Diagnostic Radiology, Department of Radiology, Faculty of Medicine, Chiang Mai University, Chiang Mai, Thailand

**Keywords:** Cardiology, Rheumatology

## Abstract

Available data including the incidence, predictors and long-term outcome of early systemic sclerosis patients associated with suspected cardiomyopathy(SSc-CM) is limited. Therefore, we aimed to study the incidence, predictors and survival of SSc-CM. An inception cohort study was conducted for early SSc patients seen at the Rheumatology Clinic, Maharaj Nakorn Chiang Mai Hospital, Thailand, from January 2010 to December 2019. All patients were determined for clinical manifestations and underwent echocardiography and HRCT at enrollment and then annually. SSc-CM was determined and classified using echocardiography. 135 early SSc patients (82 female,108 DcSSc) were enrolled. With the mean follow-up period of 6.4 years, 32 patients developed SSc-CM. The incidence of SSc-CM was 5.3 per 100-person years. The multivariate Cox regression analysis showed that baseline anti-topoisomerase I-positive (Hazard ratio[HR] 4.86, *p* = 0.036), dysphagia (HR 3.35, *p* = 0.001), CK level ≥ 500 U/L(HR 2.27, *p* = 0.045) and low oxygen saturation (HR 0.82, *p* = 0.005) were predictors of SSc-CM. The survival rates after SSc-CM diagnosis at 1, 5 and 10 years were 90.3%, 73.1%, and 56.1%, respectively. In this study cohort, the incidence of SSc-CM was 5.3 per 100-person years, and tended to have low survival. The presence of anti-topoisomerase I antibody, dysphagia, CK level ≥ 500 U/L, and low oxygen saturation were independent baseline predictors for developing SSc-CM.

## Introduction

Systemic sclerosis (SSc) is a complex autoimmune rheumatic disease, its pathogenesis associates with vascular injury, immune activation and fibroblast over proliferation. These result in tissue inflammation in the early disease phase which is then complicated by tissue fibrosis as well as fibro-occlusive vasculopathy in the later phase of the disease leading to impairment of multiple organs. Currently, cardiac complications are a prominent cause of mortality in SSc patients^1–4^. Although there has been no standard consensus regarding the definition of primary cardiac complication in SSc, it is assumed that it is a direct consequence of SSc which involves the pericardium, myocardium, conduction systems, valvular structure^5–7^ and coronary arteries^8^. Whereas, secondary cardiac complications in SSc are usually a result of pulmonary hypertension (PH) and renal involvement^6^.

Primary myocardial disease in SSc has structural abnormalities which are a consequence of multiple factors including microvascular alterations from intramural coronary vasospasm, fibro-occlusive vasculopathy, and also macrovascular coronary artery disease (CAD). However, it continues inconclusive whether CAD is contributory as a primary or secondary cardiac complication from other traditional risk factors similar to the normal population^6,9^. Poor blood supply leads to irreversible myocardial injury with typical pathologic findings such as myocardial fibrosis^10^; and immune activation leads to such typical pathologic findings as myocarditis^11,12^.

Data respecting the prevalence and incidence of SSc associated with cardiomyopathy (SSc-CM) has not been widely available due to a lack of an international, accepted definition, the variety of diagnostic methods used, and differences in study populations. Several predictors of SSc-CM have been previously reported using observational studies. These indicated the following as potential risk factors: male gender, older age, rapid skin thickening, diffuse cutaneous systemic sclerosis subtype (DcSSc), digital ulcer, vascular damage detected on nail fold capillaroscopy, myositis, pulmonary complications, tendon friction rubs, higher disease activity score, and disability index, and positive serology testing identifying anti-Scl 70, anti-histone, anti-RNA polymerase, anti-Ku, anti-Th/To and anti-U3 RNP antibodies^13–16^; and baseline CK levels ≥ 500 U/L^17^.

Currently, overall cardiac complications in SSc are often subclinical in the majority of patients^18,19^ and may result in up to one-third of SSc-related deaths^3,4,20,21^. However, published data in relation to the incidence and survival of early diagnosed SSc-CM is limited. Cardiac magnetic resonance (CMR) is becoming a highly accurate and increasingly valued tool as a non-invasive, non-irradiation exposure technique to evaluate myocardial inflammation, perfusion, and fibrosis for the assessment of SSc-CM^22^. Nonetheless, echocardiography is remaining a useful first-fundamental non-invasive imaging modality for screening and diagnosis of suspicious SSc-CM; and is generally used in clinical practice due to higher feasibility and lower cost compared with CMR.

There has been no prior published data using inception cohort study respecting incidence, predictors, and survival of SSc-CM patients determined by echocardiography. Thus, we used inception a cohort study to investigate the incidence, predictors, and survival of suspected SSc-CM determined by echocardiography in Thai patients with early diagnosed SSc.

## Patients and methods

### Patients

This study was a sub-study of the inception cohort study of the natural history of Northern Thai patients with early-diagnosed SSc managed at the Rheumatology Clinic, Maharaj Nakorn Chiang Mai Hospital, Thailand, from January 2010 to December 2019. All consecutive adult SSc patients (≥ 18 years) with a disease duration of ≤ 3 years from the first non-Raynaud’s phenomenon (NRP) contributing to SSc were enrolled^4,17^. All participants fulfilled the 1980 classification criteria of SSc^23^ and/or the 2013 ACR/EULAR classification criteria for SSc^24^. We excluded those SSc with: (i) overlapping with systemic lupus erythematosus or rheumatoid arthritis or dermatomyositis; (ii) a follow-up period of less than six months; (iii) no baseline echocardiographic or high-resolution computed tomography (HRCT) record available for review; and (iv) with underlying myocardial disease determined by echocardiography (see the definition).

### Methods

At study entry, all participants were examined for clinical characteristics, laboratory and serology^4,17^. Modified Rodnan skin score (mRSS) was used to determined severity of skin thickness^25^ and was performed by an experienced rheumatologist (S.W.). The patients then were classified as DcSSc or LcSSc subtype according to LeRoy and Medsger’s classification criteria^26^. All participants underwent electrocardiography (ECG), echocardiography, and HRCT at the baseline visit and then annually^4,17^. Echocardiographic data were reviewed by an experienced cardiologist (N.P.).

*Disease duration* was the interval from the time of the first NRP to the time of study entry^4,17^. *Follow-up duration* was the interval from the time of study entry to the time of the last follow-up or death^4,17^. The presence of *organ complication* was defined in our previously published articles including digital ulceration, gastroesophageal reflux disease (GERD), dysphagia, musculoskeletal, scleroderma renal crisis, arrhythmia, conduction defect^4,17^, and diastolic dysfunction^27^. *Muscle weakness* was defined as the presence of proximal muscle weakness and elevated CK contributed to SSc. *Interstitial lung disease (ILD)* was determined by HRCT. *Suspected pulmonary hypertension (PH*) was determined by echocardiography^28^. For more details of the study method please refer to our previously published articles^4,17^.

After eliminating patients with concomitant: (a) moderate or more severe valvular heart disease, (b) hypertensive heart disease, (c) PH, (d) CAD diagnosed by left heart catheterization, (e) hypertrophic cardiomyopathy (HCM)^29^, and (f) restrictive cardiomyopathy (RCM)^30^; the patients with structural heart abnormalities determined by echocardiography would be classified as having *suspected SSc-CM*^17^ if there is presence of *(i) dilated cardiomyopathy (DCM)*-presence of left ventricular or biventricular dilatation and systolic dysfunction in the absence of known abnormal loading conditions or significant CAD according to the 2016 European Society of Cardiology practice guideline^31^; *(ii) non-specific cardiomyopathy (nsCM)*- presence of significantly abnormal myocardial function or ventricular size not eligible for specific cardiomyopathies including CAD, DCM, HCM, and RCM^17^; *(iii) focal aneurysm-* presence of focal or segmental ventricular aneurysm, not related to CAD, without systolic dysfunction^17^; and *(iv) primary right ventricular failure*-presence of RV systolic dysfunction without suspected PH^17^.

### Statistical methods

The descriptive data are presented as frequency (%), mean ± SD, or median (interquartile range 1,3 [IQR 1,3]). Chi-square or Fisher’s exact test was used to compare the categorical variables between SSc-CM and those with non-CM. Student’s *t*-test or Mann–Whitney U test was used to compare the continuous variables between the two subgroups. The data were censored when any of the following events occurred: SSc-CM, reached the end of the study or death. The cumulative survival from the study entry was analysed using The Kaplan–Meier method. The survival between the two subgroups was compared using Log-rank test.

Multivariate Cox regression analysis with forward-stepwise selection, in which the probability of entering variables into the model was 0.15 and the probability of removing from the model was 0.2, was used to define the predictor for the evolution of SSc-CM. The variance inflation factor (VIF) and tolerance of our predictor variables were checked. *p* values < 0.05 were considered statistically significant. Statistical analyses were performed using Stata for Windows version 14.0 (TX, USA).

### Ethical issues

This study was conducted following the declaration of Helsinki and was approved by the Research Ethic Committee of Chiang Mai University (Study code: MED-2565–08851). All participants provided written informed consent at study entry.

## Results

### Demographic data

**Figure 1:**
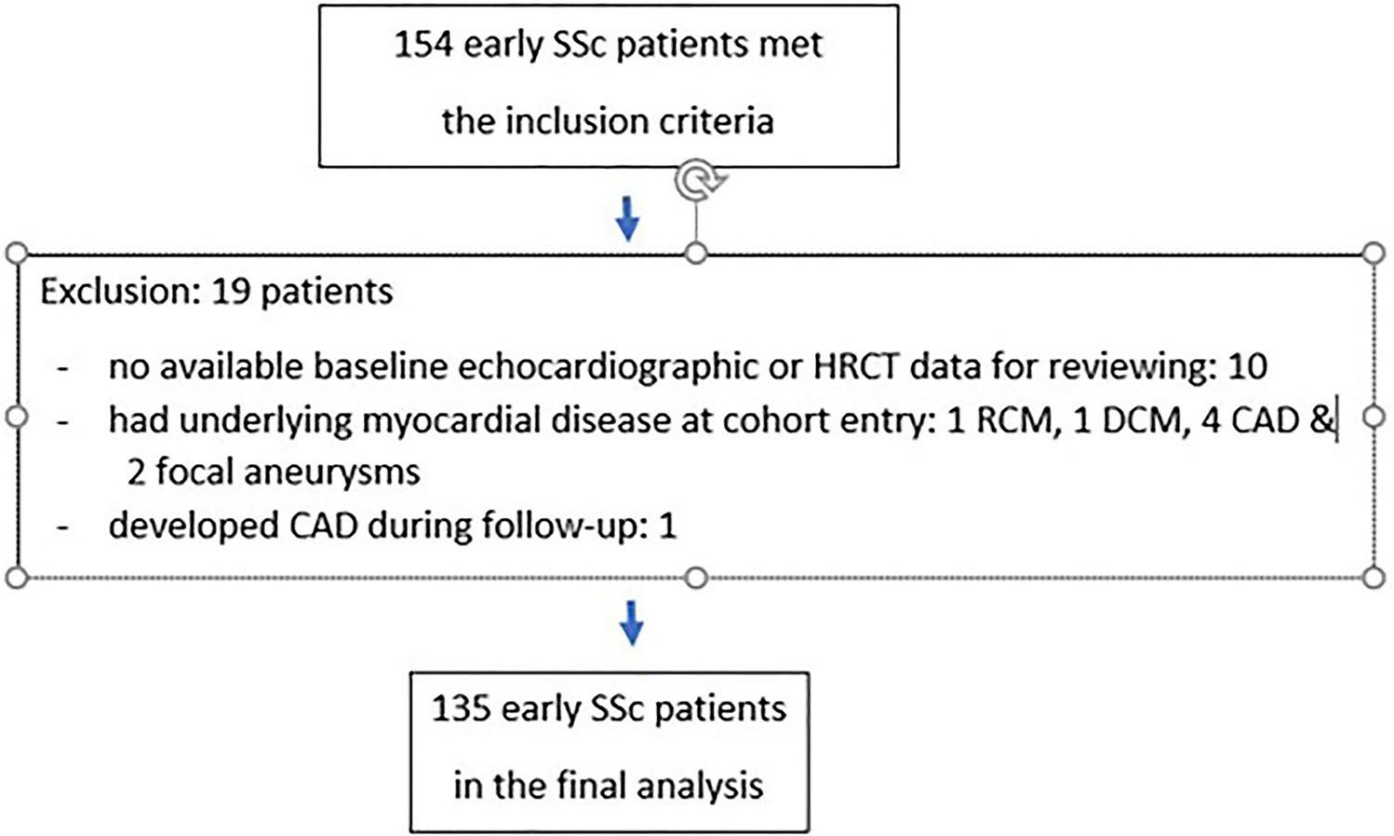
Flow diagram of study participants.

Figure 1 shows the study flow diagram of the study participants. The baseline study enrolled 154 eligible early SSc patients and 19 patients were excluded; leaving a cohort of 135 SSc patients for final analysis. Of the 135 SSc, 82 (60.7%) were female, 108 DcSSc (80.0%), 104 were anti-topoisomerase I antibody-positive (77.0%), and 11 (8.1%) anti-centromere antibody-positive. There mean ± SD age was 53.2 ± 8.9 years and disease duration were 11.6 ± 8.9 months. During the mean ± SD observational period of 6.4 ± 2.7 years, 32 of 135 SSc patients developed novel CM including 14 primary RV failures (10.4%), 10 DCM (7.4%), 6 nsCM (4.4%), and 2 focal aneurysms (1.5%). Of the 32 SSc-CM, the mean ± SD from the study entry to the diagnosis of CM was 1.9 ± 1.7 years. The incidence rate of SS-CM from the study entry was 5.3 per 100 person-years (95% CI 3.75–7.49). The participants were divided into two groups including 32 (23.7%) SSc-CM and 103 (76.3%) non-CM.

### Clinical manifestations and echocardiographic findings comparing SSc-CM patients with the non-CM group at study entry

Table 1 shows the data comparing clinical presentations, laboratory investigations and medications between the two groups at the study entry. In the SSc-CM group, there was a significant predominance of the male gender, DcSSc subtype, anti-topoisomerase I antibody-positive in comparison to the non-CM subgroup. In addition, the SSc-CM subgroup had more severe clinical presentations comprising a significant predominance of small joint contractures and dysphagia. That group also had higher mean ± SD values including mRSS, CK level, and pro-BNP level, but had lower oxygen saturation percentage than the non- CM subgroup. There were no significant differences between the two groups at enrolment concerning cardiovascular comorbidities (data not shown) such as smoking history, diabetes mellitus, dyslipidemia and hypertension; other organ complications, other laboratory results and medication received.

**Table 1:** Demographic and clinical manifestations between early SSc-CM and non-CM at study entry.

Variables	SSc-CM (n = 32)	SSc non-CM (n = 103)	*P* value
**Demographic**			
Male	18 (56.3)	35 (34.0)	**0.024**
DcSSc subtype	31 (96.9)	77 (74.8)	**0.006**
Mean age at entry, years^a^	54.4 ± 6.6	52.8 ± 9.6	0.398
Mean disease duration, months^b^	10.0 ± 7.9	12.1 ± 9.3	0.240
**Immunologic features**			
Anti-topoisomerase I antibodies	30 (93.8)	74 (71.8)	**0.010**
Anti-centromere antibodies	0	11 (10.7)	0.066
**Organ involvement**			
Digital ulcer	3 (9.4)	6 (5.8)	0.442
Telangiectasia	9 (28.1)	32 (31.1)	0.752
mRSS^b^	23.2 ± 8.5	16.9 ± 10.6	**0.001**
mRSS ≥ 18	23 (71.9)	48 (46.6)	**0.012**
Arthritis	10 (31.3)	27 (26.2)	0.577
Small joint contractures	23 (71.9)	47 (45.6)	**0.009**
Large joint contractures	9 (28.1)	19 (18.4)	0.238
Tendon friction rubs	3 (9.4)	10 (9.7)	1.000
Muscle weakness	9 (28.1)	16 (15.5)	0.109
GERD	15 (46.9)	39 (37.9)	0.363
Dysphagia	17 (53.1)	21 (20.4)	** < 0.001**
NYHA class^a^	2.1 ± 0.5	1.9 ± 0.6	0.302
% Oxygen saturation^a^	95.6 ± 2.9	96.9 ± 1.8	**0.021**
Interstitial lung disease	24 (75.0)	72 (69.9)	0.578
%pFVC (n = 16, 71)^a^	71.2 ± 15.8	73.3 ± 17.9	0.668
Suspected PH	1 (3.1)	8 (7.8)	0.686
Arrhythmia	5 (15.6)	19 (18.4)	0.715
Conduction defect	8 (25.0)	14 (13.6)	0.127
**Laboratory investigations**			
Hemoglobin, g/dL^a^	12.3 ± 1.7	12.1 ± 1.7	0.772
Creatinine, mg/dL^a^	0.8 ± 0.2	0.8 ± 0.3	0.893
CK, U/L^b^	242.5 (135.7, 868.0)	121.0 (70, 248)	**0.002**
CK ≥ 500 U/L	11 (34.4)	13 (12.6)	**0.005**
Troponin T, ng/L (n = 17, 26)^b^	16.3 (7.4, 59.3)	9.8 (5.9, 29.6)	0.224
Pro-BNP, pg./ml (n = 31, 101)^b^	288.8 (106.9, 797.1)	131.9 (56.3, 305.6)	**0.005**
ESR, mm/hr^b^	37.9 ± 27.4	40.8 ± 30.9	0.766
**Treatment**			
Prednisolone	16 (50.0)	47 (45.6)	0.665
Prednisolone dose^b^	4.1 ± 4.6	3.4 ± 4.3	0.439
Cyclophosphamide	11 (34.4)	26 (25.2)	0.312
Mycophenolate mofetil	1 (3.1)	4 (3.9)	1.000
Methotrexate	1 (3.1)	12 (11.7)	0.300
Calcium channel blocker	6 (18.8)	29 (28.2)	0.289
Phosphodiesterase-5 inhibitors	15 (46.9)	35 (34.0)	0.187
Aspirin	24 (75.0)	87 (84.5)	0.221

Values are expressed as mean ± SD, median (IQR 1,3), or n (%).

*mRSS* modified Rodnan skin score, *GERD* gastroesophageal reflux disease, *NYHA* New York Heart Association Classification, *pFVC* Predicted forced vital capacity, *PH* Suspected pulmonary hypertension, *CK* Creatine kinase, *Pro-BNP* Pro-B-type natriuretic peptide, *ESR* Erythrocyte sedimentation rate, *a* Student’s t-test, *b* Mann–Whitney U test.

Significant values are in bold.

Table 2 demonstrates the comparison of echocardiographic findings between the two groups at enrolment. SSc-CM had significantly lower left ventricular ejection function percentage (%LVEF) and larger left ventricular (LV) mass index than those without CM.

**Table 2:** Echocardiographic findings between early SSc-CM and non-CM at study entry.

Variables	SSc-CM (n = 32)	SSc non-CM (n = 103)	*P* value
% LVEF^a^	65.8 ± 7.5	68.9 ± 5.9	**0.014**
LV mass index, g/m^2^ (n = 30, 86)^a^	108.5 ± 29.6	97.6 ± 24.1	**0.046**
Peak E wave, cm/s (n = 30, 98)^a^	69.3 ± 13.5	76.2 ± 21.3	0.099
Peak A wave, cm/s (n = 30, 98)^a^	72.6 ± 17.1	73.7 ± 19.3	0.798
LV E/A (n = 30, 100)^a^	0.9 ± 0.3	1.1 ± 0.3	0.317
LV E/Em (n = 19, 71)^a^	9.8 ± 2.5	10.0 ± 3.6	0.797
LA diameter, mm (31, 103)^a^	33.0 ± 7.2	32.5 ± 6.2	0.719
RA area, mm^2^ (n = 12, 52)^a^	16.0 ± 4.4	15.3 ± 4.5	0.621
RVDd, mm (n = 22, 78)^a^	22.9 ± 3.8	21.9 ± 4.5	0.370
RVSP, mmHg (n = 26, 87)^a^	32.8 ± 7.4	33.1 ± 11.0	0.901
TR velocity, m/s (n = 28, 91)^a^	2.4 ± 0.3	2.5 ± 0.5	0.639
PA diameter, mm (n = 26, 93)^a^	22.2 ± 4.9	21.3 ± 4.8	0.437
MVDT, s (n = 28, 97)^b^	0.9 ± 3.7	0.2 ± 0.2	0.637
Diastolic dysfunction	10 (31.3)	35 (34.0)	0.775
Pericardial effusion	2 (6.3)	5 (4.9)	0.669

Values are presented as mean ± SD, or n (%).

*LVEF* left ventricular ejection fraction, *E wave*, early diastolic transmitral velocity, *A wave*, atrial filling transmitral velocity, *LA* Left atrial, *RA* Right atrial, *RVDd* Right ventricular dimension at end-diastole, *RVSP* Right ventricular systolic pressure, *TR* velocity, tricuspid regurgitation velocity, *PA* Pulmonary artery, *MVDT* Mitral valve inflow deceleration time, *a* Student’s t test, *b* Mann–Whitney U test.

Significant values are in bold.

### Predictors of SSc-CM

Table 3 shows the Cox regression analysis of predictors for SSc-CM. The univariate Cox regression analysis showed a significant association between nine baseline parameters and the development of SSc-CM (*p* < 0.05). These consisted of the male gender, DcSSc, anti-topoisomerase I antibody-positive, mRSS ≥ 18, presence of small joint contractures, muscle weakness, dysphagia, lower oxygen saturation, and CK level ≥ 500 U/L. We did not include echocardiographic parameters in the analysis for a predictor of SSc-CM as these are used for CM diagnosis. We selected those nine variables from the univariate Cox regression analysis and included them into a multivariate analysis.

**Table 3:** Predictors of SSc-CM determined by echocardiography.

Baseline characteristics	Univariate analysis			Multivariate analysis		
	HR	95% CI	*P* value	AHR	95% CI	*P* value
Male	2.30	1.14–4.63	0.020			
DcSSc subtype	8.89	1.21–65.17	0.032			
Anti-topoisomerase I	5.25	1.25–22.0	0.023	4.86	1.11–21.33	0.036
mRSS ≥ 18	2.80	1.29–6.06	0.009	1.75	0.74–4.13	0.201
Small joint contractures	2.76	1.28–5.96	0.010			
Muscle weakness	2.26	1.04–4.89	0.039			
Dysphagia	3.42	1.70–6.85	0.001	3.35	1.64–6.85	0.001
Oxygen saturation	0.79	0.69–0.90	0.001	0.82	0.71–0.94	0.005
CK ≥ 500, U/L	3.54	1.69–7.44	0.001	2.27	1.02–5.09	0.045

*AHR* Adjusted hazard ratio.

The multivariate Cox regression analysis using forward-stepwise selection for the predictors for the evolution of SSc-CM which showed the presence of anti-topoisomerase I-positive (HR 4.86, 95% CI 1.11–21.33, *p* = 0.036), dysphagia (HR 3.35, 95% CI 1.64–6.85, *p* = 0.001), and CK level ≥ 500 U/L (HR 2.27, 95% CI 1.02–5.09, *p* = 0.045) as risk factors. While the presence of higher oxygen saturation was associated with lower risk (HR 0.82, 95% CI 0.71–0.94, *p* = 0.005)**.**

### Survival of SSc-CM

At the end of the study, the SSc- CM subgroup showed a trend of higher numbers of deceased cases from all-cause deaths (9 of 32 [28.1%] vs. 22 of 103 [21.4%], *p* = 0.427) and congestive heart failure (4 of 32 [12.5%] vs. 4 of 103 [3.9%], *p* = 0.09) than the non- CM group. However, similar numbers of deceased cases from suspected PH and ILD were observed between the two groups (1 of 32 [3.1%] vs. 5 of 103 [4.9%], *p* = 1.00). There was no patient with scleroderma renal crisis in this cohort. The mean ± SD duration from diagnosis of SSc-CM to death was 2.4 ± 2.7 years. The median survival time of overall SSc-CM was 7.23 (5.9–8.5) years. The survival rate (95% CI) at 1, 5, and 10 years after diagnosis of SSc-CM was 0.90 (0.73–0.97), 0.73 (0.52–0.86), and 0.56 (0.33–0.74), respectively.

**Figure 2:**
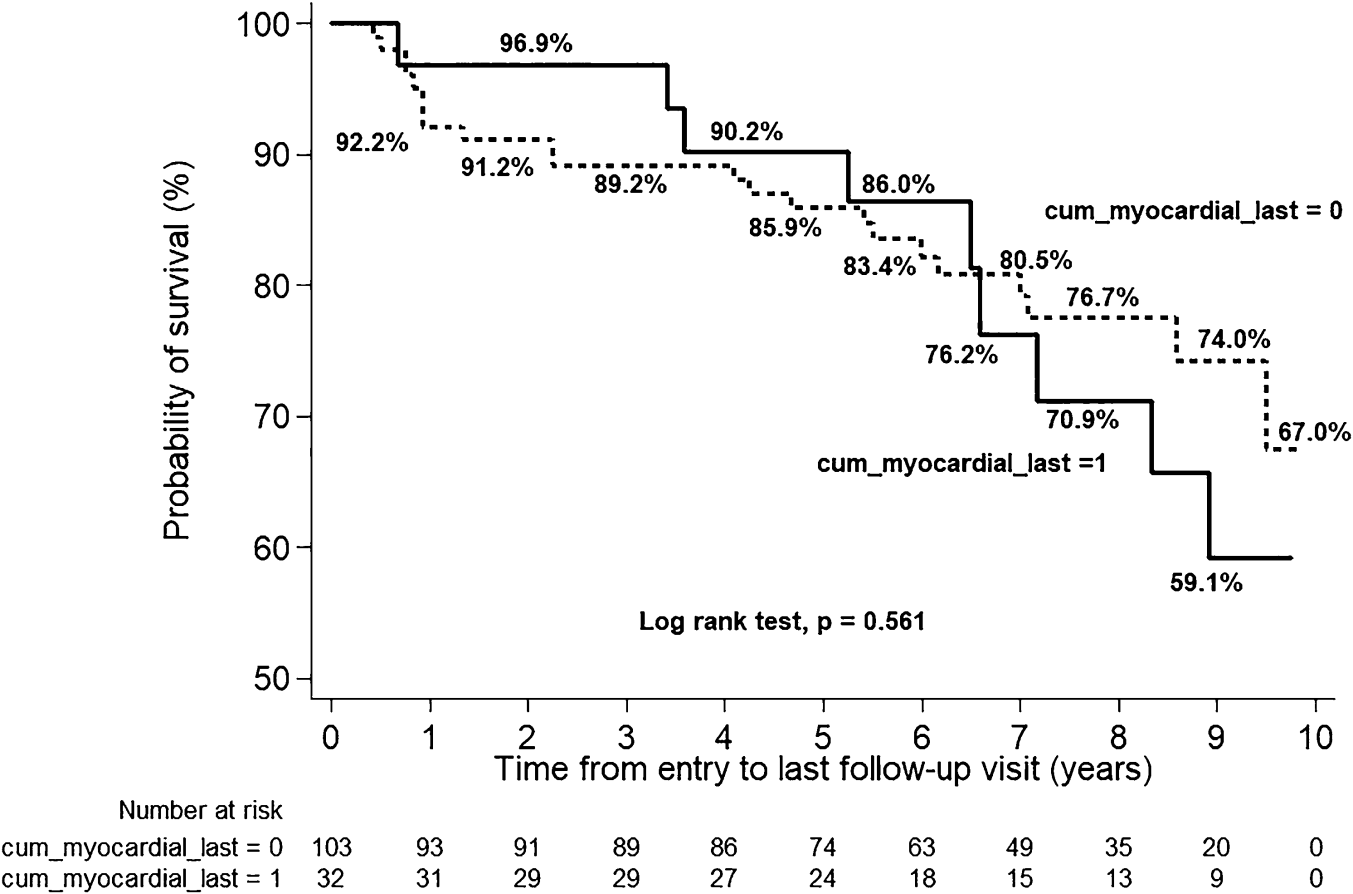
Estimated survival between SSc-CM and SSc without CM.

Figure 2 shows a comparison of the estimate survival since the study entry between the two groups analysed by the Log-rank test which showed no significant statistical difference (*p* = 0.561); however, the SSc-CM tended to have poorer survival. In the SSc-CM group, the survival rate (95% CI) from all-cause death at 1, 5, and 10 years from the study entry was 0.97 (0.79–0.99), 0.89 (0.72–0.96), and 0.56 (0.33–0.74), respectively. In the non-CM, the survival rate (95% CI) at 1, 5, and 10 years from the study entry was 0.92 (0.85–0.96), 0.85 (0.76–0.91), and 0.65 (0.52–0.75), respectively.

## Discussion

This is the first inception cohort study that reports clinical manifestations, incidence, predictors, and survival of early SSc-CM patients classified by echocardiography including DCM, nsCM, focal aneurysm, and primary RV failure. The prior reports regarding SSc-CM were cross-sectional, retrospective, or prevalence cohort studies using different definitions of SSc-CM due to a lack of globally well-accepted definitions of SSc-CM, resulting in a high degree of variation in the results^16,32^. This study cohort included early SSc patients with homogeneous mean disease duration of one year, reflecting the early phase of the disease course. In addition, Thai SSc patients had a different genetic profile from other SSc populations^33^ which manifested itself in a high prevalence of the DcSSc subtype which was anti-topoisomerase I antibody-positive with a high incidence of ILD^34^.

The incidence rate of SSc-CM found in this study from the cohort entry determined by echocardiography was modest at 5.3 per 100 person-years. Published data regarding the incidence of SSc-CM determined by echocardiography or CMR are limited. In this study, the most common echocardiographic finding of SSc-CM was primary RV failure, followed by DCM and nsCM, respectively. Further CMR study to evaluate the myocardial abnormalities in terms of myocardial edema, myocarditis, myocardial fibrosis, or subendocardial perfusion abnormalities as etiologies of the SSc-CM is needed to confirm our findings. This study also confirms that SSc-CM is not an uncommon complication in the early phases of SSc, in agreement with previous reports by Pieroni et al^35^ and Fernández-Codina A, et al.^36^ .

At enrolment, in the SSc-CM group, there was a higher prevalence of the male gender, DcSSc subtype, and anti-topoisomerase I antibody-positive. Men were found to have higher incidence of right ventricular dysfunction, ILD and more severe disease manifestations compared to women, in our prior published inception cohort data^37^. There was also more suggestive evidence of severe clinical presentation in the SSc-CM including small joint contracture, dysphagia; higher values of mRSS, CK level, and Pro-BNP level; lower values of oxygen saturation than the non-CM subgroup; and more severe left ventricular abnormalities than those with non-CM. We also found that both ILD and echocardiographic signs of SSc-CM concomitantly occurred at the first visit; however, the high prevalence of baseline ILD which was mainly a non-specific interstitial pneumonia pattern from HRCT was observed to a similar extent in both subgroups.

Predictive factors for developing SSc-CM in this early SSc cohort comprised the presence of anti-topoisomerase I antibody, which agrees with prior reports^15,16,38^. Baseline CK level ≥ 500 U/L was found to be a predictor for SSc-CM, which is concordant with prior reports^38–40^ indicating that peripheral myositis is associated with myocardial complications. We also determined the presence of dysphagia as another risk factor of SSc-CM, suggesting an association between esophageal disease and myopathy, a finding in agreement with the report by Zhou et al.^41^. Therefore, our study highlights the association between cardiac muscle, skeletal muscle, and esophageal muscle. In addition, we found that lower oxygen saturation was also a risk factor for developing SSc-CM in this cohort. A possible elucidation for this is that SSc patients with low oxygen saturation may result in poor cardiac tissue perfusion and the occurrence of myocardial injury.

With a mean observational duration of six years, the survival rate of the SSc-CM subgroup tended to be lower than the non-CM subgroup, although the difference was not statistically significant. One-third of SSc-CM patients died after a mean duration of 2.4 years following SSc-CM diagnosis, reflecting the high mortality and poor outcome.

The strength of this study was its study design as inception cohort which provides important long-term observational data in respect of incidence, predictors and survival of early SSc-CM in a specific Asian population in which the main disease subtype is DcSSc with positive anti-topoisomerase I-antibody. As the study was conducted in a single center, there was a homogeneity of clinical evaluation, echocardiography and HRCT testing that was systematically performed and recorded all through the study period.

There are some limitations in our study including: (a) a small sample size which may have decreased the power of the statistical analysis; (b) suspected SSc-CM was not determined by CMR which shows higher sensitivity and specificity than echocardiography. In addition, evaluation of troponin-T, a more specific biomarker for myocardial involvement rather than CK, was performed in only one-third of patients at baseline; (c) muscle biopsy was performed in only 29.2% of patients with CK ≥ 500 U/L to confirm myopathy associated with SSc ; (d) PH was not determined by right-sided heart catheterization; (e) only 64.4% of the participants performed pulmonary function testing at the enrolment, therefore, the severity of ILD could not be fully evaluated; (f) early immunosuppressants provided to patients may obscure the real incidence of SSc-CM. Concerning the transferability of the findings of this study, the data should be interpreted with caution because of the mean follow-up duration of around six years, suggestive of complications of the early phase of the disease.

## Conclusion

This study cohort of early SSc patients, of which the majority were of the DcSSc subtype with positive anti-topoisomerase I antibodies, has demonstrated that the incidence of SSc-CM determined by echocardiography was modest and its occurrence could develop in the first year of SSc diagnosis, resulting in a short survival time. We found that the presence of anti-topoisomerase I antibody, dysphagia, CK level ≥ 500 U/L, and low oxygen saturation were baseline-independent risk factors for developing SSc-CM in early SSc patients. Further larger prospective study regarding SSc-CM determined by CMR is crucial to confirm our findings.

## Data Availability

Data and materials are available upon request to corresponding author.
